# Large mammalian herbivores influence the deployment of microbial extracellular enzymes to control soil carbon cycling

**DOI:** 10.1007/s10533-026-01378-0

**Published:** 2026-10-06

**Authors:** Shamik Roy, Sumanta Bagchi

**Affiliations:** 1 Forest Zoology, Technische Universität Dresden, Pienner Str. 7, 01737 Tharandt, Germany; 2 Centre for Ecological Sciences, Indian Institute of Science, Bangalore, 560012 India; 3 Ecosystem Resilience Group, Institute of Evolutionary Ecology and Conservation Genomics, Ulm University, Albert-Einstein-Allee 11, 89081 Ulm, Germany

**Keywords:** Ecoeznymatic stoichiometry, Rewilding, Soil microbial enzymes, Zoogeochemistry, Carbon pump, Microbial traits, Natural climate solutions

## Abstract

Large mammalian grazers have regulated biogeochemical cycles across 40% of the Earth’s terrestrial surface for millennia. While grazer effects on organic matter entering the soil are becoming clear, their effect on the downstream microbial foraging through extracellular-enzymes (EE), which link processes at the cellular level to ecosystem-level flow of matter remain less clear. We assessed how large mammalian grazers influence microbial foraging in soil using two lines of evidence. First, utilising a 14-year-old long-term grazer-exclusion experiment in the Trans-Himalayan ecosystem, we find that activities of seven enzymes peak at different months during the growing-season in an asynchronous manner. Microbial deployment of enzymes responded to grazer-exclusion by shifting from nitrogen (N)-acquisition in spring to carbon (C)- and phosphorus (P)-acquisition in summer and autumn. Grazer exclusion increased peroxidase activity by 124.7% and β-glucosidase by 16.7%. About half of the variation in enzyme portfolios was explained by seasonal differences in grazer effects. Grazer-exclusion increased the variability in the enzyme portfolio, requiring the microbes to forage across a larger portion of the functional space. Structural equation models suggested that grazer-exclusion worsens the quality of biomass input to soil due to an 18% increase in acid-unhydrolysable recalcitrant C. As seen in other ecosystems, this decline in substrate quality raises microbial deployment of generic depolymerizers relative to the specialized enzymes which release assimilable end-products. As greater investment in generic depolymerizing enzymes may reduce microbial carbon use efficiency, it can affect the C cycle. Second, laboratory incubations supported that soil enzymes are indeed inducible/repressible in response to experimental manipulation of biomass quality. Microbial responses appear predictable and generalisable across spatial scales, from laboratory-incubations to whole ecosystems. Hence, fostering microbial functions can enhance the scope and efficacy of rewilding and restoration for natural climate solutions.

**Supplementary Information:**

The online version contains supplementary material available at https://doi.org/10.1007/s10533-026-01378-0.

## Introduction

Large mammalian grazers have been integral to the Earth’s biogeochemical cycles for millennia (Frank et al. 1998; Schmitz et al. 2018). In grasslands, shrublands, and savannas, that cover over a third of the earth’s terrestrial surface (MacDougall et al. 2026), grazers not only influence how nutrients enter and cycle between different pools, they also exert a broad influence on both above- and below-ground processes (Hobbs 1996; Frank et al. 2010; Sun et al. 2019). These functional roles of large mammalian herbivores provide ecosystem services (Yahdjian et al. 2026) and influence the Earth’s climate system (Kristensen et al. 2022; Roy and Bagchi 2022). Humans have greatly curtailed these functional roles of large grazers worldwide through hunting and land‑use change (Ripple et al. 2015; Lundgren et al. 2020). The below-ground implications of such declining influence of grazers remain unclear and inadequately inform rewilding and restoration efforts to revive ecosystem functions. In particular, it remains unclear whether grazer loss reshapes microbial foraging strategies by changing the deployment of extracellular enzymes. A variety of soil enzymes must work collectively to meet microbial nutritional requirements, and the amounts of different enzymes produced depend on spatio-temporal variation in nutrient availability and accessibility (Sinsabaugh et al. 2016). Variation in the relative production of these enzymes reflects how microbes regulate enzyme deployment through induction and de-repression (Allison et al. 2010). The ecoenzymatic costs arising from the loss of grazers can be consequential for nutrient and energy flows. Yet we still do not know how the loss of large grazers can reshape microbial foraging strategies by altering their enzyme portfolios in soil.

Grazers can define the functional space within which soil microbes forage using extracellular enzymes by altering the quantity and quality of organic matter inputs. By diverting plant biomass into secondary production and metabolism, grazers alter the quantity and quality of organic matter input to soil via dung and urine (low C:N) instead of solely senesced litter (high C:N) (Bagchi and Ritchie 2010a, b). Grazers can prevent microbes from encountering the lowest bounds of resource quality, while simultaneously restricting their access to high quantities of resources, ultimately creating a quality-vs-quantity trade-off (Bradford et al. 2016). So, grazer loss may require microbes to modify their enzyme portfolio and shift to alternative locations of the parameter space (i.e., shift in mean foraging trait). The resulting shift in resource conditions may also require microbes to forage in a smaller or larger portion of the parameter space (i.e., change in variance of foraging trait).

Shifts in microbial foraging through enzyme portfolios can have biogeochemical consequences because different extracellular enzymes carry different functional costs. Functionally, enzyme portfolios consist of (a) Generic depolymerizing enzymes: these act on large chemically complex polymers during decomposition, e.g., peroxidase, endoglucanase; (b) Specialized enzymes: these releases smaller terminal assimilable end-products for microbes (Burns and Dick 2002; Allison et al. 2010). As grazers provide steady and recurring inputs of low C:N dung and urine, one expects microbes to deploy more specialized enzymes than generic depolymerizers. But, grazer loss leads to the soil environment being inundated by high C:N litter, often all-at-once at the time of plant senescence, and one expects microbes to deploy more generic depolymerizers than specialized assimilatory enzymes (Roy and Bagchi 2022). Here, altering the enzyme portfolio by microbes can affect fundamental metabolic properties such as their carbon use efficiency (CUE). Since CUE links processes at the cellular scale with ecosystem-level material and energy flow (Burns and Dick 2002; Falkowski et al. 2008) grazer loss can have broad implications for biogeochemical cycles and the climate system (Schmitz et al. 2014; Reinhart et al. 2022; Naidu et al. 2022).

Here, we investigate how large mammalian grazers regulate microbial foraging and their enzyme portfolios in soil. We posit two primary hypotheses: (1) we predict that the loss of grazers alters both the mean and variance of the parameter space used by microbes for foraging, where microbes shift their enzyme portfolio away from specialized nutrient acquisition and toward generic-depolymerizing enzymes; and (2) this grazer-induced shift, in the stoichiometry of specialized to generic depolymerizing enzymes, which increases the demand for depolymerizing enzymes, would decrease microbial CUE. A shift towards producing generic depolymerizing enzymes may require microbial investment of C and energy, thereby reducing CUE. This is because microorganisms must invest more resources in extracellular processing before assimilable products become available. We utilized a long-term, 14-year, grazer-exclusion field experiment in the Trans-Himalayan grazing ecosystem of northern India to address seasonal and functional trajectories of the soil microbiome and its enzyme portfolios. Concurrently, we used laboratory incubations to target the upper and lower ends of resource availability for microbes as senesced litter and as dung to experimentally quantify the credible bounds of functional plasticity in microbial enzyme deployment. By bridging these two approaches, we aim to address how the functional roles of large mammalian grazers extend below ground. Specifically, we examined whether trade-offs in resource quality and quantity influence microbial foraging in soil.

## Materials and methods

We combined a long-term field grazer-exclusion experiment with a laboratory manipulation experiment to investigate whether and how large mammalian grazers regulate microbial foraging. We used the field study was used to quantify seasonal variation in microbial EE activities. In the laboratory experiment, we tested whether changes in substrate quality could induce short-term changes in enzyme activity.

### Study site

Our study site in Spiti, northern India (32° N, 78° E), is a high-altitude grazing ecosystem in the Trans-Himalaya which is a part of the Tibetan highlands and adjacent mountains in Central Asia. This ecosystem is highly seasonal, with temperatures falling below −30 °C in winters; daytime temperatures reach 15 °C in spring, peaking near 25 °C during summer, and dropping again in autumn (Fig. S1). Annual precipitation of c. 450 mm yr^−1^ is in the form of snow (100–200 cm) during November-March and rain (150–300 mm) during July-August (Fig. S1). The vegetation growing season (Fig. S1) is short and extends from May to August with peak near mid-July. By August-September, plants begin to senesce, and litter detritus gets buried under snow and ice during the winter months from November to March. Temperatures become favorable again for high levels of plant and microbial activity after spring snowmelt in April onwards. Aboveground net plant production is c. 50 g m^−2^, of which about 60% is consumed during the growing season by a diverse assemblage of large mammalian grazers (Bagchi and Ritchie 2010a, b; Bagchi et al. 2017). The grazer assemblage comprised of native species and domesticated livestock that includes goat, sheep, donkey, horse, ibex, bharal, cattle, yak, and yak-cattle hybrids. Vegetation at our study site consists of grasses from the genera *Poa*, *Elymus*, *Festuca*; sedges from *Carex*, *Kobresia*; forbs from *Lindelofia*, *Astragalus*; and shrubs from *Artemisia*, *Caragana* (Bagchi and Ritchie 2010b, a; Baidya et al. 2025).

### Sampling

We established 30 grazer-exclusion plots starting in 2005-06, each 100 m^2^ (10 × 10 m), with a paired-adjacent control plot. The vegetation composition did not change between fenced and grazed plots (Baidya et al. 2025). We collected soil samples at monthly intervals during the growing season between May and September 2017, with a 5 cm diameter corer to a depth of 20 cm. We removed stones and pebbles with a 2 mm sieve and stored soils in sterilized vials for transport and subsequent laboratory analyses. We analyzed enzyme activity within 4–5 days from the time of collection (Roy and Bagchi 2022).

### Soil and microbial variables

Soil C content ranges between 1.5–2.0%, whereas soil N ranges between 0.1–0.2% (Roy and Bagchi 2022). Geologically, the soils are derived from uplifted ocean beds and are coarse-textured (sandy-loam), with approximately 70–95% sand and comparatively low silt and clay contents (Fig. S2). Soil pH ranged from near neutral to slightly alkaline (Fig. S2). Water-holding capacity ranged approximately from 0.8 to 1.3 mL g^−1^, and bulk density ranged from 1.0 to 1.45 g cm^−3^ (Fig. S2).

We measured the activities of five hydrolytic and two oxidative enzymes. The enzymes are C-acquiring β-glucosidase and cellobiohydrolase, N-acquiring leucine aminopeptidase and *N*-acetyl-β-D-glucosaminidase, P-acquiring phosphatases, generic depolymerizing peroxidase, and multipurpose polyphenol oxidase. Six of these enzymes are directly involved in foraging – one is a generic depolymerizer, and five are specialised enzymes (Table S1). The seventh enzyme, polyphenol oxidase, is a multi-purpose enzyme because it is not only involved in decomposition and microbial foraging but also in non-foraging functions; e.g., defence mechanisms (Sinsabaugh 2010). Briefly, we homogenized 1 g soil in 100 mL of Tris buffer at pH 7.8, as this is close to soil pH, to prepare a suspension. We prepared enzyme-specific substrates and added them to the suspension (summary in Table S1, 200 mM for Fluorogenic substrates and 25 mM for chromogenic substrates, 0.3% H_2_O_2_). We used a fluorescent standard of 10 µM. We conducted assays on a microplate; 200 µL of substrate was added to 50 µL of soil slurry for hydrolytic enzymes, whereas for oxidative enzymes 50 µL of substrate was added to 200 µL of slurry, with 10 µL of H_2_O_2_ added for peroxidase assays. Incubation time was 2 h for hydrolytic enzymes and 15 h for oxidative enzymes. We incubated all slurries at 20 °C, as this approximates average prevailing temperature in the study site during the growing season. Except for oxidative enzymes, we stopped the reactions by adding 10 µL of 2M NaOH at the end of the incubation period and measured the fluorescence within 1 minute of the addition. For fluorogenic measurements, we used a 96-well fluorimeter (Tecan Infinite® M200 Pro, Mannedorf, Switzerland) at excitation wavelength 365 nm and emission wavelength 450 nm. For colorimetric reactions, we transferred 100 µL of reaction mixture to 96-well plates and measured absorbance at 460 nm. For internal quality checks, we replicated each sample for all assays four times with corresponding standards, and then averaged (median) the readings before statistical analysis of enzyme activity (nmol h^−1^ g^−1^ soil) (Saiya-Cork et al. 2002; German et al. 2011). We calculated mass-specific enzyme activity as the ratio of enzyme activity (above) and microbial biomass C.

We measured microbial biomass C (MBC) and microbial biomass N (MBN) using chloroform fumigation-extraction (Anderson and Domsch 1978). Briefly, we incubated 4 g of soil at 60% water holding capacity with ethanol-free chloroform for 24 h, extracted in 0.05 M K_2_SO_4_, and analyzed for C content and N content in TOC analyzer (Shimadzu, TOC-L, Japan). We calculated MBC (mg C g^−1^ soil) and MBN (mg N g^−1^ soil) as the difference between fumigated and paired unfumigated samples. We considered an extraction efficiency of 0.45 for MBC and 0.54 for MBN (Beck et al. 1997).

We estimated fungal and bacterial contributions to microbial biomass by selectively inhibiting fungi with the antifungal antibiotic cycloheximide and bacteria with the antibacterial antibiotic streptomycin (Anderson and Domsch 1975; Bagchi et al. 2017). Briefly, for fungal and bacterial contributions, we incubated 4 g soil at 60% water holding capacity with trace amounts of glucose (0.5% w/w) and respective antibiotics (8 mg g^−1^ soil) alongside controls (without antibiotics). We measured CO_2_ respired from soil samples after 24 h of incubation using an alkali trap (1N KOH), followed by titration with acid (0.1N HCl, after adding 15% BaCl_2_). Fungal and bacterial contribution to microbial biomass was estimated as the difference in CO_2_ respired with respect to controls (Fig. S3). We estimated the relative contribution of fungi and bacteria as $\mathrm{l}\mathrm{n}\left(\frac{Fungal {CO}_{2}}{Bacterial {CO}_{2}}\right)$, where positive values indicate fungal dominance and negative values indicate bacterial dominance (Anderson and Domsch 1975; Bagchi et al. 2017).

We used two-step acid hydrolysis to measure labile/recalcitrant pools (biochemical quality) in soil organic matter, hereafter SOM (Rovira and Vallejo 2002; Rovira and Ramón Vallejo 2007; Khalili et al. 2016). The first pool is labile and acid-hydrolysable, and mainly consists of hemicellulose and non-cellulosic polysaccharides derived either from plants or microbes. The second acid-hydrolysable pool, or the intermediate pool, is primarily cellulose. The difference between total organic C and the sum of labile C and intermediate C was judged to be recalcitrant C that remains unhydrolyzed (Rovira and Vallejo 2002; Rovira and Ramón Vallejo 2007; Khalili et al. 2016). Briefly, in soil organic matter, we added 20 mL of 5 N H_2_SO_4_ to 500 mg of soil, and incubated for 30 min at 105 °C. After incubation, we centrifuged the mixture at 3000 RPM for 2 min (1000 × g force approx., Remi R-4C, Bangalore) and stored the supernatant as the labile pool. To the remaining soil residue, we added 2 mL of 26 N H_2_SO_4_ and incubated overnight with continuous shaking. After incubation, we diluted the acid to 2 N by adding water, and incubated again at 105 °C for 3 hours. This was also centrifuged at 3000 RPM for 2 min, and the supernatant was stored as the intermediate pool. We measured C content in a TOC analyzer (Shimadzu, TOC-L, Japan). Each fraction was expressed as mg g^−1^ soil.

We estimated apparent CUE based on the ecological stoichiometry of the microbes, where the CUE depends on the elemental requirements (C and N) for growth and the elemental composition of the substrates in the environment that will support the organism’s growth (Sinsabaugh et al. 2013, 2016). We estimated CUE from stoichiometric relationships between MBC and MBN, labile C and N, and C-acquiring enzyme (β-glucosidase) and N-acquiring enzyme (leucine aminopeptidase and *N*-acetyl-β-D-glucosaminidase) in soil (Sinsabaugh et al. 2013, 2016). Microbial CUE consists of two interrelated aspects of their metabolism. First, assimilation of available C substrates from the environment into biomass requires a short-term response, which is measured using stable isotopic tracers. This is used as a proxy for intrinsic CUE. In environmental samples, however, C may undergo multiple cycles of microbial uptake, release, and reuse that cannot be resolved by community-level estimates. Second, microbes utilize these assimilated substrates to fulfil their metabolic demands. This utilization pattern relates to long-term stoichiometric relationships between microbes and soil C, and is interpreted as apparent CUE. Hereafter, we refer to apparent CUE as CUE. It is measured through the activity of their extracellular enzymes and is more appropriate for our questions (Schimel et al. 2022). We calculated CUE following this equation: $E=\frac{{CUE}_{\mathrm{m}\mathrm{a}\mathrm{x}} \times S}{S + k}$, where $S=\frac{1}{{Enzyme}_{\mathrm{C}:\mathrm{N}}}\times \frac{{Biomass}_{\mathrm{C}:\mathrm{N}}}{{Labile}_{\mathrm{C}:\mathrm{N}}}$, where *k* = 0.5 is half-saturation constant, with *CUE*_max_ = 0.6 is the upper limit of microbial CUE (Sinsabaugh et al. 2013).

### Inducibility and/or de-repression of enzyme

We tested the hypothesis on plasticity of microbial production of enzyme in response to the upper and lower end points of biomass-quality. To test this hypothesis, we conducted a laboratory incubation with reciprocal transplants of senesced litter as a relatively low-quality, high-C:N substrate litter and dung as a relatively high-quality, pre-digested, low-C:N substrate. We considered litter to be of low quality since the average C:N of litter from our study site is 60:1, whereas dung is higher in quality with C:N of 40:1 (Bagchi and Ritchie 2010a, b). We verified whether experimentally manipulating the quality of biomass input indeed initiates a short-term physiological response from soil microbes (i.e., functional plasticity in enzyme portfolios as a microbial trait), by measuring soil enzymes. The reciprocal-substrate experiment used soil collected from n = 6 paired plots in August 2019. First, we checked whether the enzyme profiles in August 2019 were similar to August 2017 as verification of initial conditions (i.e., Day-0). We found that enzyme profiles were not different between samples from 2017 and 2019 (PERMANOVA, F_1,21_ = 0.004, p = 0.97). Next, we incubated 5 g of soil sample from each plot at 20 °C, representing typical daytime temperature in mid-summer and at 60% water holding capacity to provide a non-water-limiting but aerobic incubation environment (Iovieno and Bååth 2008) under the following treatments/control conditions:

- Control group 1. No additional substrate was added to monitor baseline enzyme profiles.
- Control group 2. These soils received their “home” substrate. 0.5 g dry senesced litter was added to soil from fenced plots, and 0.5 g of dry dung (an equal mixture of dung from yak, yak-cattle hybrid, bharal, cattle, goat, donkey) was added to soils from grazed plots. Each microcosm received 0.5 g of substrate per 5 g of soil, corresponding to a 10% substrate addition by soil mass. The loading was selected as a standardised experimental pulse that was large enough to elicit a measurable response while retaining soil as the dominant microcosm component.
- Treatment. These soils received “away” substrate. 0.5 g litter was added to soils from grazed plots to reduce the quality of biomass input. 0.5 g dung was added to soils from fenced plots to increase the quality of biomass input.

We measured the effect of change in the quality of biomass input as a difference in enzyme activities between the treatment and control group 2. We measured enzyme profiles for all groups on the 2nd, 4th, and 8th day (Fig. S4). We incubated the soil samples in triplicate for each soil sample and each of the three treatment/control conditions. We collected soil from six paired fenced and grazed plots, giving 12 independent source soils. The design therefore contained 12 source soils × three substrate conditions × three harvest times, for a total of 108 soil microcosms, plus additional substrate-only controls. On the 2nd, 4th, and 8th day of the incubation, we would take out one of the triplicates from the incubation and measure the enzyme activity in the soil. During each destructive harvest, the soil and substrate within each microcosm were thoroughly mixed, and a representative subsample of the combined matrix was taken for enzyme analysis. Using independent microcosms for each harvest ensured that samples collected on days 4 and 8 had not been disturbed by earlier sampling.

We had a substrate-only control, where we measured the enzyme activity in only dung and litter to estimate the contribution of substrates towards enzyme activity in soil for the reciprocal transplant experiment. These measurements were made at the start of the experiment. We found enzyme activities were much higher in dung than in litter, except for P-acquiring phosphatase, which was higher in litter than in dung (Fig. S5).

### Statistical analyses

#### Univariate analysis of responses to grazer-exclusion

To distinguish whether microbes regulate enzymes selectively through the seasons (selective deployment strategy), or whether they produce all the enzymes equally at all times (a *laissez-faire* strategy), we evaluated the synchrony between the enzymes with Kendall’s τ. Strong positive correlations would indicate that enzymes tended to increase and decrease together, whereas weak or inconsistent correlations would indicate asynchronous allocation. We used linear mixed-effects models (GLM) with repeated measures to evaluate how grazer-exclusion and months influence the soil variables. We assessed gross and mass-specific extracellular enzyme activities (i.e., enzyme activity/microbial biomass), soil C pools (labile, intermediate, and acid-unhydrolysable recalcitrant), microbial biomass, fungal:bacterial biomass ratios (F:B), and CUE as response variables. We included grazing treatment (fenced vs. grazed), month (May through September), and their interaction as fixed effects, while plot identity (n = 30 pairs) was as a random effect to account for the paired experimental design. We utilized a first-order autoregressive (AR1) correlation structure to account for temporal dependencies between the months. We summarized the direction and magnitude of responses in grazer-exclusion as the log-response ratio at each paired plot, $\Delta =\mathrm{l}\mathrm{n}(Fenced/Grazed)$, for these variables. The grazing × interaction may reflect both short-term seasonal changes in animal use and legacy effects accumulated during the long-term exclusion treatment.

#### Multivariate analysis

We assessed the responses to grazer-exclusion and changing seasons in enzyme portfolios using a combination of ordination and dispersion analyses. First, to address the question of differences in mean foraging trait, we used constrained ordination with Redundancy Analysis (RDA). RDA assessed and helped visualize shifts in enzyme portfolios and checked whether the observed variability in the enzyme portfolios is explained by the grazing × season interaction. Second, to address variance of functional traits, we quantified the multivariate dispersion of the enzyme portfolios as Bray-Curtis dissimilarity, with Cailliez correction before Principal Coordinates Analysis (PCoA). We then calculated distances to group centroids using betadisper in the vegan package (R 4.1.0). We compared the distance to centroids for each treatment × month combination (PERMDISP) with 999 permutations using the permutest function.

#### Microbial functional placticity & structural equations model

The reciprocal-transplant laboratory incubations aimed to assess the upper and lower bounds of microbial functional plasticity in enzyme portfolios. Responses to the two endpoints of resource quality, senesced litter and dung, indicate if soil enzymes are inducible in response to experimental shifts in substrate quality. We tested for differences in enzyme activity using a GLM with change in substrate quality and enzyme identity as fixed effects. Next, we used a mixed-effects structural equations model (SEM) to evaluate the evidence supporting hypothesized causal paths that link grazer-exclusion to microbial CUE via their enzymes. We retained the paired (fenced/grazed plots) data structure in the SEM by incorporating plot identity and growing season as random effects. SEM paths were motivated by *a priori* instances known in literature (Bagchi et al. 2017; Roy and Bagchi 2022; Roy et al. 2023b, a). After the full model, we simplified the SEM by dropping paths for enzymes C2, N1, N2, P and Mp, since they explained the least variation (Roy and Bagchi 2022). To avoid circular tautologies, we did not include paths from the labile fraction of SOM and from microbial biomass in the SEM, as CUE was derived from these variables. Similarly, we did not model a path from the constituent enzymes to CUE. We assessed the agreement between data and hypothesized paths with Fisher’s C statistic in piecewiseSEM package (Shipley 2009; Lefcheck 2016). We used directed-separation tests and did not find any missing paths in our model. All analyses were performed in R 4.1.0 using the nlme and piecewiseSEM libraries.

## Results

**Fig. 1:**
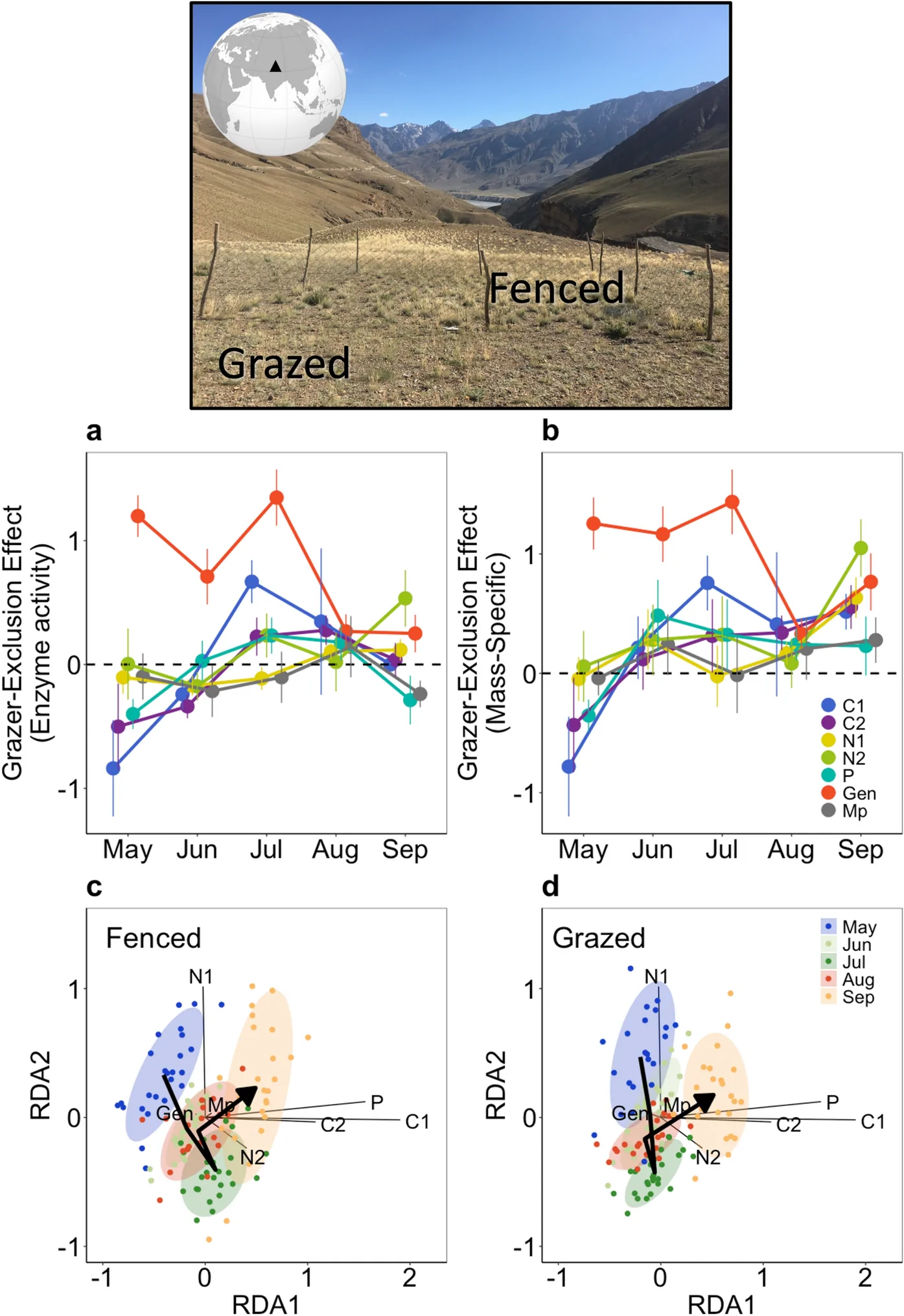
Grazer-exclusion effect on enzyme activity, as log-ratio of paired fenced/grazed plots, in (**a**). Grazer-exclusion effect on mass-specific enzyme activity in (**b**). Seasonal shift in enzyme composition in fenced (**c**) and grazed (**d**) plots, visualised through Redundancy analysis (RDA). Shift in trajectories of enzyme with season is marked for the groups’ centroids. Loss of grazers increases investment in generic depolymerizers, while suppressing investment in specialized enzymes, and their effect is stronger early in the growing season, but they have a weak effect on the multi-purpose enzyme. Data in c and d are from n = 30 paired plots (grazed and fenced) in Spiti, northern India ($\overline{x } \pm SE$). Abbreviations – C1: specialized C-cycle enzyme β-glucosidase, C2: specialized C-cycle enzyme cellobiohydrolase, N1: specialized N-cycle enzyme leucine aminopeptidases, N2: specialized N-cycle enzyme *N*-acetyl-β-D-glucosaminidase, P: specialized P-cycle enzyme phosphatase, Gen: generic depolymerizer peroxidase, and Mp: multi-purpose enzyme polyphenol oxidase

We find that gross as well as mass-specific enzyme activities showed pronounced asynchrony across the months, as different enzymes peaked at different points in the growing season (Fig. 1a, b, Fig. S6, S7). These functional trajectories of enzymes were significantly affected by grazer-exclusion (Fig. 1c, d, RDA: Grazing × Season interaction F_9,188_ = 21.04, p < 0.001), explaining 50.6% of total variation in enzymes. Specifically, grazer-exclusion shifted the enzyme portfolio away from specialized nutrient-acquiring enzymes and toward generic depolymerizers. Gross peroxidase activity (generic depolymerizer) was 124.7% higher in fenced plots (F_1,137_ = 39.37, p < 0.001), particularly in the early half of the growing season. Similarly, fenced plots showed increased investment in specialized enzymes, including 16.7% higher gross β-glucosidase (F_1,137_ = 5.07, p = 0.026), relative to grazed controls. Multipurpose polyphenol oxidase was not different between fenced and grazed plots. These effect sizes were broadly consistent between gross and mass-specific enzyme activity (Table S2). Crucially, grazer-exclusion affected the variability of the enzyme portfolios since multivariate dispersion was higher in fenced plots compared to grazed plots (PERMDISP: F_9,239_ = 3.02, p = 0.002). Higher dispersion in fenced plots suggests that grazer loss requires microbes to forage across a larger portion of the functional parameter space.

**Fig. 2:**
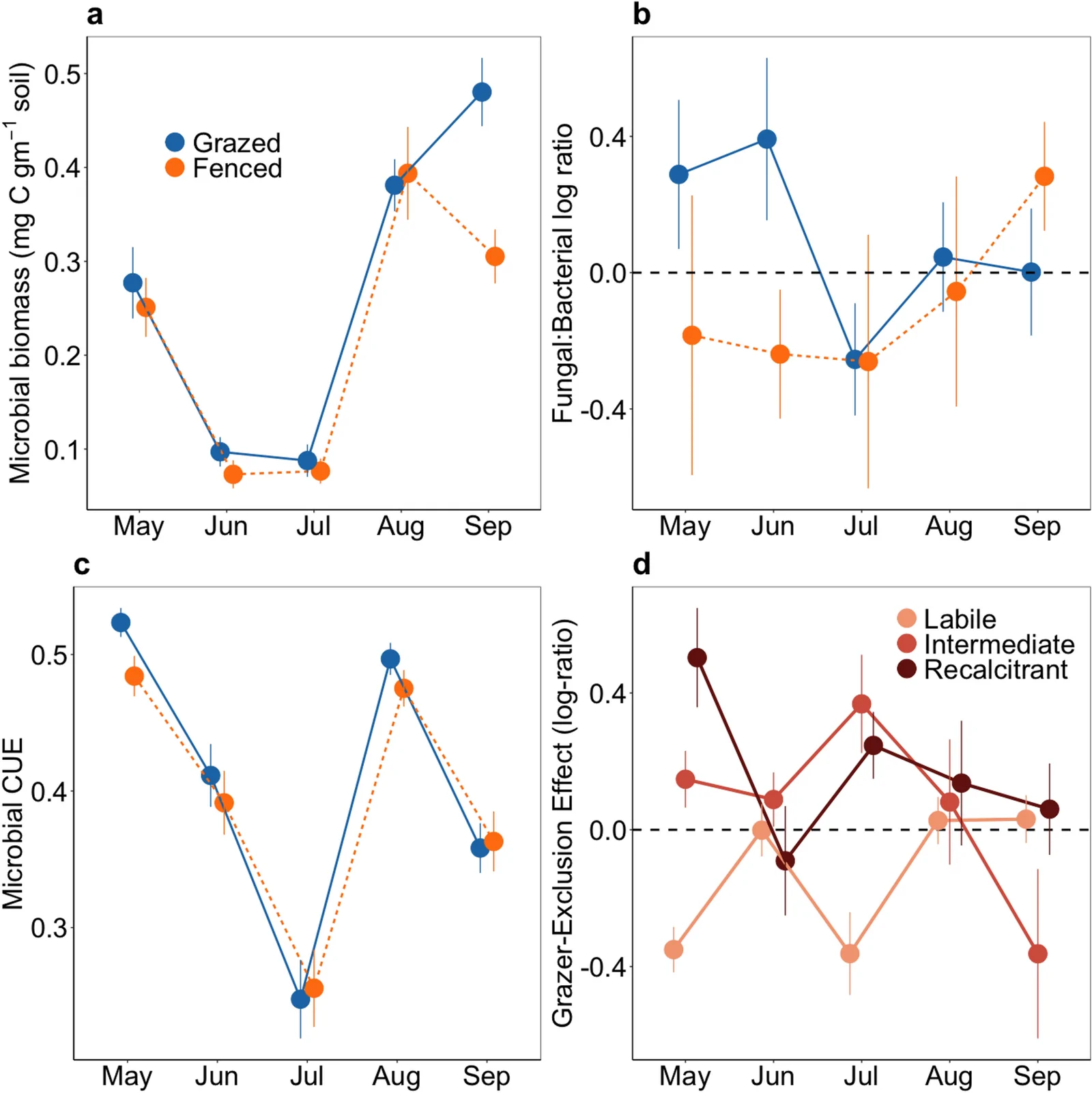
Seasonal trajectories of microbial and soil variables that suggest decoupling between microbial community composition of microbial communities and their functional attributes. Seasonal variation in microbial biomass (**a**), Fungal:Bacterial ratio (**b**), and microbial Carbon Use Efficiency (CUE) **c** in grazed and fenced plots. The grazer-exclusion effect on soil C pools is summarized as log-ratio of paired fenced and grazed plots (**d**). Direction and magnitude of differences in biomass, fungal:bacterial ratio, and C pools varied with grazing and time. Data ($\overline{x } \pm SE$) are from n = 30 paired plots (grazed and fenced) in Spiti, northern India

Beyond enzymatic shifts, grazer-exclusion also reconfigured the metabolic and structural aspects of the soil microbial environment. Microbial biomass was 18.9% lower in fenced plots (Fig. 2a, Table S3), where grazer-exclusion favored a bacterial-dominated community due to reduced F:B specifically during the early growing season. These changes reflected a broader shift in resource biochemical quality; fenced plots exhibited a 10.7% decline in labile soil C alongside 6.7% higher intermediate pool and 18% higher acid-unhydrolysable recalcitrant C pool (Fig 2d, S8, Table S3). Such altered resource conditions indicate a measurable metabolic cost, in the form of a 7% reduction in microbial CUE, in fenced plots during the early season.

**Fig. 3:**
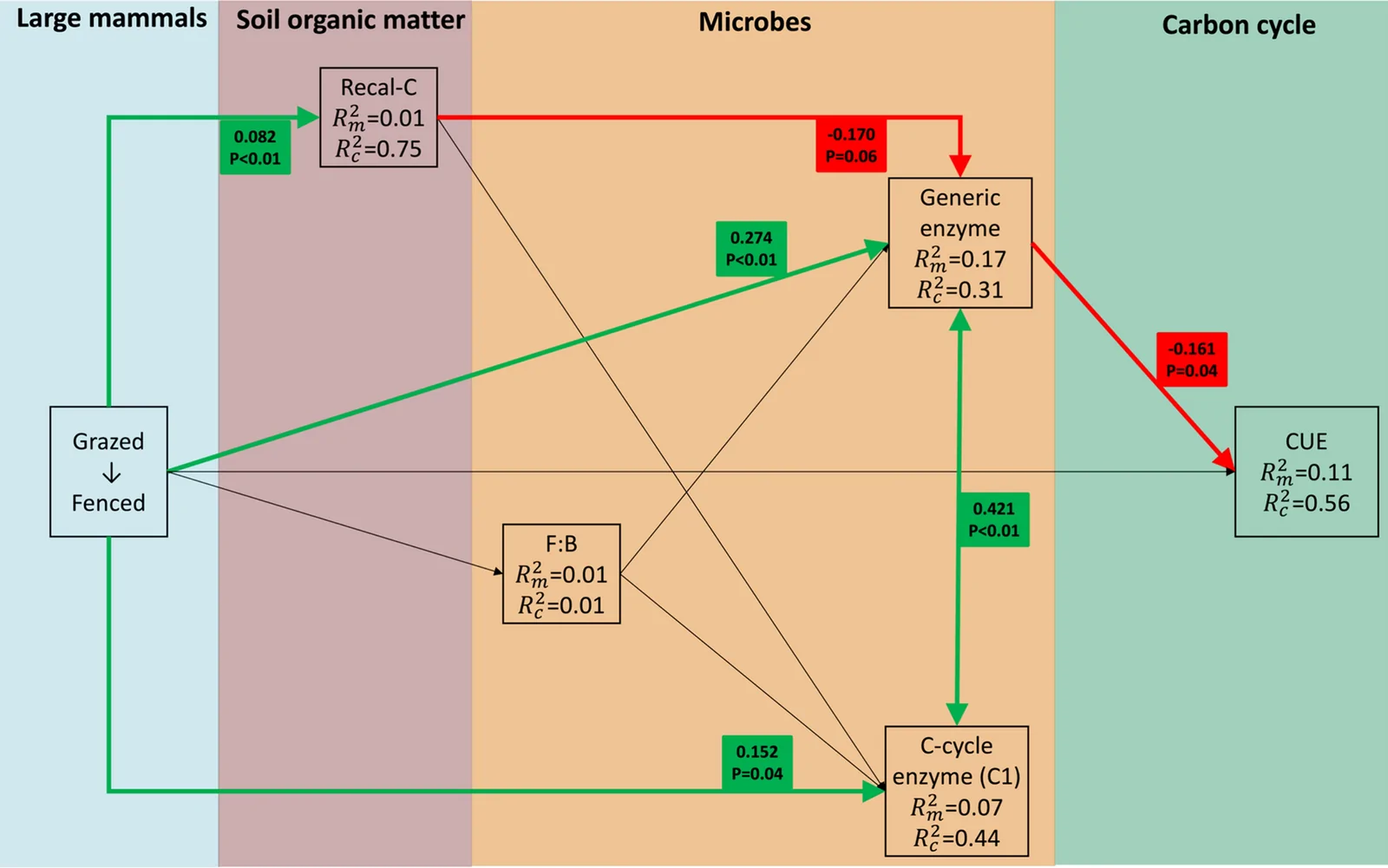
Summary of mixed-effects structural equation modelling to evaluate how grazing by large mammalian grazers affects microbial foraging strategy via the quality (changes in labile and recalcitrant pools) of soil organic matter, ultimately influencing the C cycle. SEM paths show grazer-exclusion alters microbial foraging to reduce microbial CUE. Unidirectional arrows represent hypothesized causal paths, and bidirectional arrows indicate correlated paths. Thick green (positive path) and red (negative path) arrows are statistically significant paths; thin black arrows are statistically non-significant paths (α = 0.05). Standardized path coefficients and their statistical significance are shown alongside marginal and conditional R^2^ for the different variables. Data are from n = 30 permanent paired plots in Spiti, northern India. Abbreviations – Recal-C: recalcitrant C, F:B : fungal-bacterial biomass ratio, C1: specialized C-cycle enzyme β-glucosidase, Generic enzyme: generic depolymerizer peroxidase

Structural equations model (SEM) confirmed the hypothesized link between grazer-exclusion and ecosystem-scale C cycle (Fisher’s C = 9.94, *p* = 0.127, df = 6, Fig. 3, Fig. S9). The paths suggest that grazer-exclusion affects the soil C pool through both direct and indirect pathways. This is because excluding grazers increases the acid-unhydrolysable recalcitrant fraction of soil organic matter, which in turn elevates the deployment of generic depolymerizing enzymes. Notably, grazer-exclusion exerted a significantly stronger influence on C cycle enzymes than on N cycle enzymes, primarily through heightened investment in peroxidase. This community-wide shift in foraging traits can manifest as a critical metabolic bottleneck as it reduces microbial CUE. These paths support the expectation that grazers regulate ecosystem processes via microbial foraging strategies at the cellular and community levels.

**Fig. 4:**
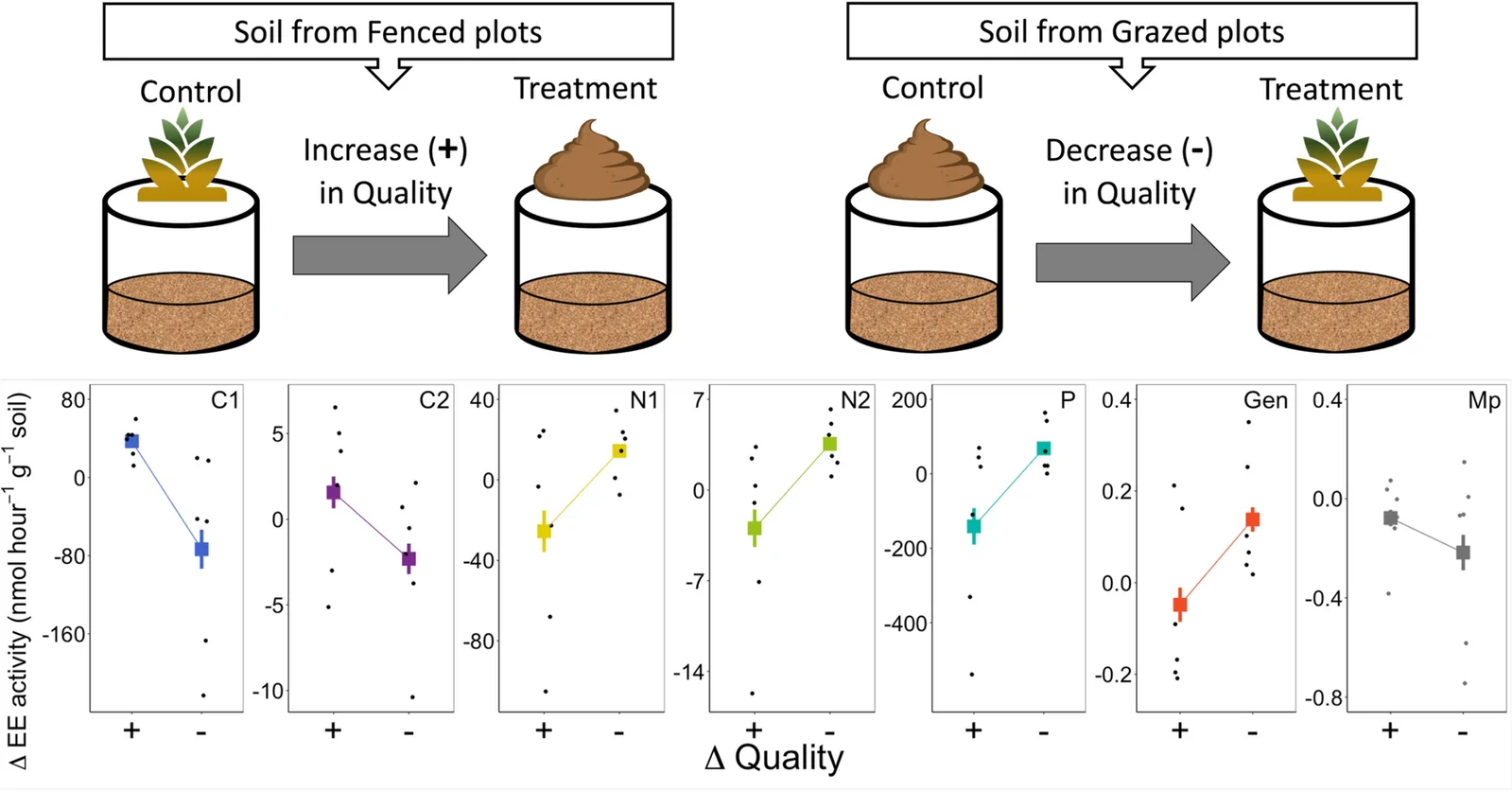
Experimental validation of microbial response to change in biomass quality (C:N). “Increase (+) in quality” represents soil from fenced plots treated with dung (high quality or low C:N), calculated relative to the corresponding fenced soil receiving its home senesced litter substrate (low quality or high C:N). “Decrease (-) in quality” represents soil from grazed plots treated with senesced litter, calculated relative to the corresponding grazed soil receiving its home dung substrate. The enzyme panel below shows the change (Δ) in enzyme activity calculated as the difference between the “treatment” group (home-substrate) and the “control group 2” (away substrate). Data are from n = 6 paired plots (grazed and fenced) in the semi-arid grazing ecosystem in Spiti region, northern India ($\overline{x } \pm SE$), after 4 days of incubation. Abbreviations are as in Fig. 1. Microbial foraging response to biomass-quality is inducible

The laboratory reciprocal transplant experiment validated that the microbial foraging shifts observed in the field were a direct and inducible response to changes in resource quality (Fig. 4, Table S3). The endpoints of the resource continuum, litter-only and dung-only, established the credible bounds of microbial functional plasticity (Fig. 4, Table S3). Experimentally lowering substrate quality (litter addition) initiated a rapid induction of generic depolymerizing enzymes (peroxidase), mimicking the functional state of fenced plots in the field. Conversely, improving resource quality through dung suppressed generic enzyme production and shifted the enzyme portfolio toward specialized enzymes, as previously seen in the grazed plots in the field. Multipurpose enzymes (polyphenol oxidase) remained unresponsive to these manipulations. The absence of a multipurpose enzyme response is consistent with its broader functions beyond resource acquisition. Microbial EE response varied with the incubation time (Table S3). On day 2, the change in substrate quality alone had an overall significant effect on EE activity (Table S3). This response became stronger on day 4, when a change in substrate quality had a different effect on the seven EE due to a significant substrate-quality × enzyme interaction (Table S3). By day 8, the overall effect of substrate quality had weakened, although enzyme-specific responses persisted (Table S3). These results indicate a rapid but enzyme-dependent response rather than a uniform directional change in total enzyme activity. Collectively, these results demonstrate that microbial enzyme portfolios vary predictably as they are responsive to changes in quality of biomass inputs brought about by grazer-exclusion.

## Discussion

Our results show that long-term grazer-exclusion altered both the seasonal composition and the dispersion of microbial extracellular-enzyme portfolios. Below, we first discuss the evidence for seasonal enzyme allocation, then evaluate potential mechanisms underlying the grazing response, and finally consider the implications for soil C and N processing.

### Do microbes deploy a foraging strategy and do large mammalian grazers influence it?

Enzyme activity showed clear signatures which are consistent with a foraging strategy; not all enzymes were equally active in the soil at the same time. This asynchrony in enzyme production is not explained by variation in microbial biomass (Fig. 2). Here, we can infer that microbes do not invest equally in all enzymes. Instead, they are selective in deploying enzymes through the growing season (Fig. 1a–d). The patterns indicated a focus on N acquisition early in the season during spring, whereas microbial enzyme allocation shifted towards C and P acquisition later during summer and autumn (Fig. 1c, d). Such directional shifts are consistent with reports from other ecosystems, such as temperate savanna, arctic tundra and tropical forests (Boerner et al. 2005; Wallenstein et al. 2009; Schaap et al. 2023).

We find that grazer-exclusion has a strong effect on microbial foraging, with the activity of the peroxidase being high in fenced plots (Figs. 1, 3, S9). Grazer-exclusion, together with seasons, explained half the variation in enzyme activity. Overall, the SEM indicates that grazer-exclusion has a stronger effect on the C-cycle enzymes than on N-cycle enzymes (Fig. 3, S9). The apparently stronger response of C-cycle enzymes reflects the absence of significant direct and indirect paths from grazer exclusion to the measured N-acquiring enzymes (Fig. S9), and resultantly, these paths were muted in the global SEM as well (Fig. 3). Large mammalian grazers influenced microbial deployment of extracellular enzymes by shifting the average enzyme profile, and by constraining the breadth of functional space occupied across the growing season. Grazing restricts microbes to a narrower region of functional space, whereas grazer-exclusion expands the range of enzyme combinations expressed through time. Thus, the loss of grazers does not simply move microbial decomposers to a different average foraging state; it broadens the parameter space over which they must forage.

### How do large herbivores influence microbial enzyme activities?

Since grazer effects on gross and mass-specific enzyme activity were similar (Fig. 1a, b), the observed differences in enzyme activity were not an artefact of change in microbial biomass through time (Fig. 2). The difference in enzyme residence times also does not explain the difference between paired grazed and fenced plots (Sinsabaugh 2010). So, we explored whether observed differences could be due to shifts in the microbial community composition (Malik et al. 2016), as grazing is known to favor fungal dominance over bacteria in high altitude grazing ecosystems (Chen et al. 2015; Bagchi et al. 2017). We found fungal dominance to be higher in the grazed plots, especially early in the growing season (Fig. 2). Since peroxidase is primarily secreted by fungi and less by bacteria (Sinsabaugh 2010), microbial community shifts alone do not explain the observed patterns in enzyme activities. Instead, these patterns may reflect how the existing community responds to changes in quality of biomass input to soil (i.e., dung vs litter).

Temporal variation in the resources of soil microbes is both a cause and a consequence of their enzymatic actions. As the growing season progresses, the availability of resources as labile/recalcitrant soil C changes (Fig. 2d, Fig. S8). Grazing primarily influenced the acid-unhydrolysable recalcitrant pool, and relative availability of recalcitrant C peaked during the middle of the growing season in the fenced plots, in comparison to the grazed plots (Fig. 2d, Fig. S8). The rise in the acid-unhydrolysable recalcitrant pool indicates incorporation of litter accumulated from autumn and winter into soil organic matter as temperature becomes favourable in spring and summer (Djukic et al. 2018). This organic matter is then transformed by microbial processes, and the peak in acid-unhydrolysable recalcitrant C coincided with a peak in generic depolymerizer enzyme in fenced plots (Fig. 1). Importantly, the SEM recapitulates the change in quality of nutrient input to soil due to grazer-exclusion. Indeed, studies across shrub-steppes, arctic meadows, temperate prairies, conifer savannas, alpine steppes, and temperate woodlands (Stritar et al. 2010; Stark et al. 2012; Francini et al. 2014; Cline et al. 2017; Moorhead et al. 2017; Santalahti et al. 2018; Dong et al. 2020; Feng et al. 2025) have also found that grazer-exclusion increases the activity of the generic depolymerizer enzyme in soil based on one-time measurements. We find that with repeated sampling across the growing season, grazer exclusion changes both the average enzyme profile and the range of enzyme combinations expressed through time. Thus, the microbial response to biomass-quality appears to be both widespread and general. In fact, animal effects on microbial resources may not be restricted to only large mammalian grazing systems. For example, wintering crane activity increased microbial biomass while reducing plant-derived C inputs (Min and Choi 2022). Together with our results, this comparison suggests that animal-mediated changes in the quantity and chemical form of organic inputs can reorganise below-ground microbial activity, although the direction and mechanism will depend on animal identity, vegetation and soil conditions.

Long-term grazing can alter microbial activity indirectly through soil compaction, water retention or pH. However, bulk density, water-holding capacity and pH did not differ significantly between grazed and fenced plots in our study (Fig. S2). Differences in these measured soil properties are therefore unlikely to be the primary explanation for the observed enzyme responses, although other unmeasured physical changes in soil structure cannot be excluded.

### Can shifts in microbial enzymes alter the C-cycle?

The paths in our SEM confirm that grazer-exclusion drives predictable shifts in enzyme portfolios. The identified associations help address the mechanisms underlying the patterns seen in previous studies from the Trans-Himalaya and other ecosystems around the world (Schmitz et al. 2014; Reinhart et al. 2022; Naidu et al. 2022). The SEM indicates that grazer-exclusion shifts microbial enzyme portfolios toward greater investment in generic depolymerizing enzymes, especially peroxidase, under low-quality substrates. Greater relative investment in generic depolymerizing enzymes may represent a costlier mode of microbial foraging, because accessing C from recalcitrant organic matter requires greater extracellular investment before assimilable products become available. As a result, grazer-exclusion is associated with lower microbial CUE, where a larger fraction of C is lost through respiration rather than incorporated into biomass (Schmitz et al. 2014; Reinhart et al. 2022; Naidu et al. 2022). This C could have accumulated in fairly stable organo-mineral complexes through a more efficient C pump (Bradford et al. 2016; Cotrufo et al. 2019). Thus, the loss of grazers can reduce soil C storage and stability by altering microbial foraging through extracellular enzymes.

Grazers divert much of the above-ground plant biomass away from the litter pathway. When in their rumen, this plant material is acted upon by a cohort of enzymes which provide energy to both the grazer and the gut microbes (Selinger et al. 1996; Forsberg et al. 1997; Solden et al. 2018). As in soil, the enzymes of rumen microbes consist of generic depolymerizers that act on higher order polymers, and specialized enzymes that act downstream to yield terminal assimilable end-products (Burns and Dick 2002). But energetic constraints over gut-retention time imply that grazers cannot retain the digesta indefinitely (Belovsky 1997; Hammer et al. 2017), and it must be periodically egested as dung. Microbial and enzymatic processing during passage through the digestive system of grazers partially breaks down structural plant polymers (Susmel and Stefanon 1993), and this semi-degraded matter is returned to soil as dung (Sankaran and Augustine 2004; Bagchi et al. 2017). We see that enzyme activities are much higher in dung than in soil or litter (Fig. S5). From the soil microbes’ perspective, grazers and rumen microbes alter low-quality litter into high-quality dung. The increase in low-quality litter requires microbes in the fenced plots to use more generic depolymerizers (Roy and Bagchi 2022), when compared to grazed plots (Fig. 1). Additionally, grazing can have a similar effect as N addition, such that dung and urine in the grazed plot offset the N limitation for the microbes to lower the deployment of peroxidase. Importantly, multi-purpose polyphenol oxidase, which is not a primary foraging enzyme, seems relatively uninfluenced by grazer-exclusion (Fig. 1). A subsidy arising in the rumen over metabolically expensive peroxidase can result in net energetic gains for soil microbes (Sinsabaugh 2010). This subsidy may be viewed as a form of mutualism between the two sets of microbiota, where each attempts to use plant-derived polymers as their energy source – once in the rumen, and again in the soil (Roy and Bagchi 2022). Soil microbes may return the favor by accelerating rates of nutrient cycling, which promotes compensatory growth among grazed plants (Bagchi and Ritchie 2011). This increased nutrient availability ultimately improves foraging opportunities for grazers (Ritchie et al. 1998; Bagchi and Ritchie 2010a; Geremia et al. 2025).

### Enzymes in relation to quality and quantity of biomass input

If microbes indeed forage selectively in response to quality of biomass input to soil, then enzyme portfolios should respond to experimental changes in biomass quality. We validated this argument through experimental manipulations by incubating soils from fenced plots with dung, and soils from grazed plots with litter (i.e., a reciprocal transplant; Fig. 4, Fig. S5). When microbes from soils under high-quality dung (grazed plots) were given low-quality litter, they secreted more generalized enzymes, and more N- and P-acquisition enzymes. In contrast, microbes produced less C-acquisition enzymes (Fig. 4). When microbes from soils under low-quality litter (fenced plots) are given high-quality dung, they respond in the opposite direction (Fig. 4). An increase in generic depolymerizer with a decrease in quality of biomass input to soil reflects much higher inputs of recalcitrant C from litter (Roy and Bagchi 2022). Increased P-acquiring enzyme activity suggests high P investment towards cellular machinery (Margalef et al. 2017). An increase in N-acquiring enzyme activity with a decrease in substrate quality indicates relative N limitation arising from the high C:N of the litter input to soil, leading to increased N mining (Rousk et al. 2016; Kristensen et al. 2018). Remarkably, a decrease in C-acquisition enzyme activity with a decrease in biomass input quality reflects the overlapping consequences of low-quality biomass input. When soil microbes encounter low-quality biomass, they invest proportionately more in generic depolymerizers than in specialized C-acquisition enzymes. A similar pattern is observed in fenced plots after herbivore exclusion. The response of EE to experimental manipulation of substrate quality validates that grazing by large mammalian herbivores alters the quality of biomass input to soil, which in turn initiates a predictable response in how soil microbes forage. In reality, a grazing ecosystem does not operate at the two extremes, litter-only and dung-only. By continuously subsidizing the soil with high-quality inputs (dung, improved litter quality), large mammalian herbivores prevent the system from entering the lower credible limit of pure litter inputs. Consequently, grazers sustain the microbial community within a buffered range of conditions and regulate the C cycle via CUE by preventing extreme fluctuations in biomass input.

### Conclusions and future directions

From a microbial perspective, the loss of large mammalian grazers in the Trans-Himalayan grazing ecosystem has stronger effects on the C cycle that arise from responses in generic depolymerizing oxidative and C-acquiring enzymes, than on the N cycle, which ultimately reduces CUE. The question now arises, whether this microbial response to the loss of large mammalian grazers is general and widespread. The contrasting responses of C and N enzymes can lead to an imbalance in the rates of C and N cycling. Since, these microbial responses are generalizable across scales, i.e., from lab incubations to whole ecosystems, human impact on ecosystems are fundamentally altering how microbes process nutrients. Fostering microbial processes, that have evolved over millennia, through rewilding and restoration of large mammalian grazers can enhance the opportunities for natural climate solutions.

## Supplementary Information

Below is the link to the electronic supplementary material.

Supplementary file1 (DOCX 1828 kb)

## Data Availability

The data has been uploaded to FigShare with https://doi.org/10.6084/m9.figshare.28789964
