# Supplementary material for: Large mammalian herbivores influence the deployment of microbial extracellular enzymes to control soil carbon cycling
Source: Biogeochemistry. 2026 Oct 6;169(5):62. doi: 10.1007/s10533-026-01378-0 (PMC13642401; doi:10.1007/s10533-026-01378-0)
Supplement: Supplementary file 1 [file 10533_2026_1378_MOESM1_ESM.docx]

Journal: Biogeochemistry

Manuscript type: Article

**Supplementary Information for**

**Large mammalian herbivores influence the deployment of microbial extracellular enzymes to control soil carbon cycling**

Shamik Roy^1, 2, 3^ *, Sumanta Bagchi^2^

1-Technische Universität Dresden, Forest Zoology, Pienner Str. 7, 01737 Tharandt, Germany

2-Centre for Ecological Sciences, Indian Institute of Science, Bangalore 560012, India

3-Ecosystem Resilience Group, Institute of Evolutionary Ecology and Conservation Genomics, Ulm UniversityAlbert-Einstein-Allee 11, 89081, Ulm, Germany

This Supplementary Information provides supporting figures and full statistical results for the field grazer-exclusion experiment and reciprocal dung–litter incubation. The supplementary figures show seasonal climatic conditions, microbial responses, individual enzyme trajectories, enzyme synchrony, incubation responses and structural equation models. Together, these materials support the manuscript’s evaluation of how long-term grazer exclusion alters seasonal microbial enzyme allocation and associated soil properties.

**Contents**:

Fig. S1. Seasonality of temperature and precipitation in the study area

Fig. S2. Summary of abiotic variables

Fig. S3. Temporal variation in fungal and bacterial biomass

Fig. S4. Enzyme-activity on Day-2 and Day-8 of reciprocal-transplant experiment

Fig. S5. Enzyme-activity in dung and litter

Fig. S6. Temporal variation in activities of seven soil microbial extracellular enzymes

Fig. S7. Correlation between different enzymes

Fig. S8. Seasonal variation in three pools of soil organic matter

Fig. S9. SEM for enzyme

Table S1. List of EE

Table S2, S3. Summary of GLM

Fig. S1: Description of strong seasonality in Spiti region of northern India in terms of precipitation (**a**), temperature (**b**), and plant biomass as satellite-derived normalized difference vegetation index, NDVI (**c**) in the last 5 years (2013-2017). Precipitation occurs primarily in the winter months, with a lesser contribution from the South Asian summer monsoon. Temperature is favourable for plant growth after during May-August. Consequently, vegetation growth begins in May, reaches peak biomass in July-August, and senesces thereafter. The growing season is depicted in grey. Temperature data are from MODIS 1-km resolution land surface temperature (LST), precipitation data are from CHIRPS monthly 5.5-km resolution and NDVI data are from 30-m resolution LANDSAT-7.

Fig. S2. Different abiotic variables across sampling locations in the study area. Data are from *n*=30 plots in Spiti, Trans-Himalaya.

Fig. S3: Temporal variation in substrate-induced respiration (SIR) when supplemented with glucose only (**a**), glucose plus antifungal antibiotic cycloheximide (**b**), and glucose plus antibacterial antibiotic streptomycin (**c**). Data are from n=30 paired plots (grazed and fenced).

Fig. S4: Change in seven enzyme-activities (mean ± SE) with respect to corresponding controls on day-2 and day-8 after the start of reciprocal transplant experiment. Change in enzyme-activity varied according to identity of the enzyme and change in substrate quality for both day-2 (GLM, F_6,63_=22.61, P<0.001), and day-8 (GLM, F_6,62_=4.26, P=0.001). Effect of change in quality of biomass-input was initiated at day-2, was strongest on day-4 (Fig. 3), and faded by day-8. Data are from n=6 paired plots (grazed and fenced). This verifies that experimental change in quality of biomass-input results in a short-term physiological response from soil microbes. Names are abbreviated as in Fig. 1.

Fig. S5: Activities of seven soil microbial extracellular enzymes (mean ± SE) in dung and litter collected from Spiti region of northern India for reciprocal transplant experiment as substrate. Different enzyme activities varied according to substrate (dung and litter; GLM, F_6,42_=26.89, P<0.0001). Overall, enzyme activities were higher in dung than in litter. Names are abbreviated as in Fig. 1.

Fig. S6: Temporal variation in activities of seven soil microbial extracellular enzymes in Spiti region of northern India. Different enzyme-activity varied according to season and grazing (GLM, F_24,1813_=2.11, P=0.001). Data (mean ± SE) are from n=30 paired plots (grazed and fenced).

Fig. S7: Correlation (Kendall’s) between different soil microbial extracellular enzymes from Spiti region of northern India. Correlation of enzyme activity (**a**) and mass-specific enzyme activity (**b**). Overall, enzyme activities are not synchronous and are broadly uncorrelated through time. Blank cells indicate correlations that were not statistically significant (P ≥ 0.05). Names are abbreviated as in Fig. 1.

Fig S8. Seasonal variation among labile (**a**), intermediate (**b**), and recalcitrant (**c**) carbon fractions of soil organic matter in Spiti region of Northern India. The different C-pools varied according to season and grazing (GLM (C-pool × Season × Grazing), F_8,790_=3.83, P<0.001). Data (mean ± SE) are from n=30 paired plots (grazed and fenced).

Fig S9: Summary of mixed-effects structural equation modelling to evaluate how grazer-exclusion affects each enzyme. Unidirectional arrows represent hypothesized causal paths, and bidirectional arrows indicate correlated paths. Thick green and red arrows are statistically significant paths; thin black arrows are statistically non-significant paths (α = 0.05). Standardized path coefficients and their statistical significance are shown alongside marginal and conditional R^2^ for the different variables.

Table S1**:** Description of the enzymes. The target molecule represents the nutrient against which the enzyme is secreted in the soil. IUBMB classification is the nomenclature of the enzymes based on the reactions they catalyse. Substrate used to measure the enzyme-activity are mostly fluorogenic, except substrates for Mp and Gen. IUBMB is abbreviation for International Union of Biochemistry and Molecular Biology ([www.sbcs.qmul.ac.uk/iubmb/](http://www.sbcs.qmul.ac.uk/iubmb/)).

| Name | Target | Abbreviation | IUBMB Class | Substrate |
| --- | --- | --- | --- | --- |
| Beta-Glucosidase | Carbon | C1 | 3.2.1.21 | 4-MUB β-D- glucopyranoside |
| Cellobiohydrolase | Carbon | C2 | 3.2.1.91 | 4-MUB β-D -cellobioside |
| Leucine Aminopeptidase | Nitrogen | N1 | 3.4.11.1 | L-Leucine-7-amido-4-methylcoumarin hydrochloride |
| Beta-*N*-acetylglucosaminidase | Nitrogen | N2 | 3.2.1.52 | 4-MUB N-acetyl-β-D- glucosaminide |
| Phosphatase | Phosphorous | P | 3.1.3.1 | 4-MUB phosphate |
| Polyphenol Oxidase | Multipurpose | Mp | 1.10.3.1  1.10.3.2  1.13.11.1  1.13.11.2  1.14.18.1 | L-dihydroxyphenylalanine (DOPA) |
| Peroxidase | Generic | Gen | 1.11.1.7  1.11.1.13  1.11.1.14 | L-dihydroxyphenylalanine (DOPA) &  Hydrogen peroxide |

Table S2**:** Detailed results from general linear models for analysis of variation in seven extracellular enzymes. Results are from GLM with the grazer-exclusion effect as the main effect, time (5 months) and exclosure id (30 exclosures) as random effects, applied to each of the gross enzyme activities and to the mass-specific enzyme activity.

| Gross or mass specific | Variable | F | P |
| --- | --- | --- | --- |
| Gross | Beta-Glucosidase | F_1,137_=5.07 | 0.026 |
|  | Cellobiohydrolase | F_1,137_=0.32 | 0.571 |
|  | Leucine Aminopeptidase | F_1,137_=0.21 | 0.649 |
|  | Beta-*N*-acetylglucosaminidase | F_1,137_=0.37 | 0.545 |
|  | Phosphatase | F_1,137_=1.37 | 0.243 |
|  | Polyphenol Oxidase | F_1,137_=0.01 | 0.938 |
|  | Peroxidase | F_1,137_=39.37 | <0.0001 |
|  |  |  |  |
| Mass specific | Beta-Glucosidase | F_1,137_=3.63 | 0.058 |
|  | Cellobiohydrolase | F_1,137_=0.01 | 0.935 |
|  | Leucine Aminopeptidase | F_1,137_=0.12 | 0.731 |
|  | Beta-*N*-acetylglucosaminidase | F_1,137_=0.09 | 0.765 |
|  | Phophatase | F_1,137_=2.17 | 0.143 |
|  | Polyphenol Oxidase | F_1,137_=1.29 | 0.258 |
|  | Peroxidase | F_1,137_=21.10 | <0.0001 |

Table S3**:** Detailed results from general linear models for analysis of variation in different variables. Results are from GLM for grazer-exclusion effect on enzyme activity (Fig1. a), grazer-exclusion effect on mass specific enzyme activity (Fig1. b), microbial biomass (Fig2. a), fungal/bacterial ratio (Fig2. b), microbial carbon use efficiency (Fig2. c), grazer-exclusion effect on soil carbon pools (Fig2. d), change in enzyme activity on day-4 of reciprocal transplant experiment (Fig4.), change in enzyme activity on day-2 of reciprocal transplant experiment (Fig. S3), change in enzyme activity on day-8 of reciprocal transplant experiment (Fig. S3), raw values of enzyme activity in dung and litter (Fig. S4), raw values of enzyme activity in grazed and fenced plots (Fig. S5), and raw values of different soil carbon pools in grazed and fenced plots (Fig. S7).

| Figure | Variable | Effect | F | P | *R^2^* Marginal | *R^2^* Conditional |
| --- | --- | --- | --- | --- | --- | --- |
| Fig1. **a** | Grazer-exclusion effect (Enzyme Activity) | Time | F_4,894_=5.48 | <0.001 | 0.15 | 0.16 |
|  |  | Identity | F_6,894_=15.33 | <0.0001 |  |  |
|  |  | Time×Identity | F_24,894_=2.99 | <0.0001 |  |  |
|  |  |  |  |  |  |  |
| Fig1. **b** | Grazer-exclusion effect (Mass specific) | Time | F_4,894_=2.61 | 0.034 | 0.09 | 0.10 |
|  |  | Identity | F_6,894_=15.62 | <0.0001 |  |  |
|  |  | Time×Identity | F_24,894_ =2.48 | <0.001 |  |  |
|  |  |  |  |  |  |  |
| Fig2. **a** | Microbial Biomass | Time | F_4,237_=82.73 | <0.0001 | 0.49 | 0.60 |
|  |  | Grazing | F_1,237_=9.75 | 0.002 |  |  |
|  |  | Time×Grazing | F_4,237_=4.69 | 0.001 |  |  |
|  |  |  |  |  |  |  |
| Fig2. **b** | Fungal/Bacterial ratio | Time | F_4,172_=1.65 | 0.16 | 0.06 | 0.07 |
|  |  | Grazing | F_1,172_=1.69 | 0.19 |  |  |
|  |  | Time×Grazing | F_4,172_=2.08 | 0.08 |  |  |
|  |  |  |  |  |  |  |
| Fig2. **c** | Microbial Carbon Use Efficiency (CUE) | Time | F_4,210_=49.52 | <0.0001 | 0.43 | 0.46 |
|  |  | Grazing | F_1,210_=0.97 | 0.326 |  |  |
|  |  | Time×Grazing | F_4,210_=0.50 | 0.733 |  |  |
|  |  |  |  |  |  |  |
| Fig2. **d** | Grazer-exclusion effect (carbon pools) | Time | F_4,394_=1.03 | 0.391 | 0.09 | 0.10 |
|  |  | Carbon pool | F^­­^_2,394_=5.78 | 0.003 |  |  |
|  |  | Time×Carbon pool | F_8,394_=3.85 | <0.001 |  |  |
|  |  |  |  |  |  |  |
| Fig4. | Δ Enzyme Activity | Change in substrate quality | F_1,65_=1.67 | 0.20 | 0.29 | 0.34 |
|  |  | Identity | F_6,65_=0.45 | 0.84 |  |  |
|  |  | Change in substrate quality × Identity | F_6,65_=5.35 | <0.001 |  |  |
|  |  |  |  |  |  |  |
| Fig. S4- Day2 | Δ Enzyme Activity | Change in substrate quality | F_1,58_=43.97 | <0.0001 | 0.77 | 0.80 |
|  |  | Identity | F_6,58_=16.06 | <0.0001 |  |  |
|  |  | Change in substrate quality × Identity | F_6,58_=24.05 | <0.0001 |  |  |
|  |  |  |  |  |  |  |
| Fig. S4- Day8 | Δ Enzyme Activity | Change in substrate quality | F_1,57_=3.46 | 0.068 | 0.33 | 0.43 |
|  |  | Identity | F_6,57_=1.60 | 0.164 |  |  |
|  |  | Change in substrate quality × Identity | F_6,57_=4.88 | <0.001 |  |  |
|  |  |  |  |  |  |  |
| Fig. S5 | Enzyme activity (Raw Values) | Substrate | F_1,42_=0.22 | 0.640 | *R^2^* = 0.993 | *R^2^* adj= 0.991 |
|  |  | Enzyme | F_6,42_=230.95 | <0.0001 |  |  |
|  |  | Substrate×Enzyme | F_6,42_=26.89 | <0.0001 |  |  |
|  |  |  |  |  |  |  |
| Fig. S6 | Enzyme activity (Raw Values) | Time | F_4,1833_=139.85 | <0.0001 | 0.69 | 0.70 |
|  |  | Grazing | F_1,1833_=0.09 | 0.75 |  |  |
|  |  | Identity | F_6,1833_=320.38 | <0.0001 |  |  |
|  |  | Time×Grazing | F_4,1833_=3.96 | 0.003 |  |  |
|  |  | Time×Identity | F_24,1833_=47.26 | <0.0001 |  |  |
|  |  | Grazing×Identity | F_6,1833_=2.44 | 0.02 |  |  |
|  |  | Time×Grazing× Identity | F_24,1833_=2.69 | <0.0001 |  |  |
|  |  |  |  |  |  |  |
| Fig. S8 | Carbon pools  (Raw Values) | Time | F_4,817_=29.05 | <0.0001 | 0.27 | 0.42 |
|  |  | Grazing | F_1,817_=2.29 | 0.130 |  |  |
|  |  | Identity | F_2,817_=6.29 | 0.002 |  |  |
|  |  | Time×Grazing | F_4,817_=0.63 | 0.639 |  |  |
|  |  | Time×Identity | F_8,817_=26.50 | <0.0001 |  |  |
|  |  | Grazing×Identity | F_2,817_=7.16 | <0.001 |  |  |
|  |  | Time×Grazing× Identity | F_8,817_=3.25 | 0.001 |  |  |

References

Anderson JPE, Domsch KH (1975) Measurement of bacterial and fungal contributions to respiration of selected agricultural and forest soils. Can J Microbiol 21:314–322. https://doi.org/10.1139/m75-045

Bagchi S, Roy S, Maitra A, Sran RS (2017) Herbivores suppress soil microbes to influence carbon sequestration in the grazing ecosystem of the Trans-Himalaya. Agric Ecosyst Environ 239:199–206. https://doi.org/10.1016/j.agee.2017.01.033

Beck T, Joergensen RG, Kandeler E, et al (1997) An inter-laboratory comparison of ten different ways of measuring soil microbial biomass C. Soil Biol Biochem 29:1023–1032. https://doi.org/10.1016/S0038-0717(97)00030-8

Khalili B, Ogunseitan OA, Goulden ML, Allison SD (2016) Interactive effects of precipitation manipulation and nitrogen addition on soil properties in California grassland and shrubland. Applied Soil Ecology 107:144–153. https://doi.org/10.1016/j.apsoil.2016.05.018

Rovira P, Vallejo VR (2002) Labile and recalcitrant pools of carbon and nitrogen in organic matter decomposing at different depths in soil: an acid hydrolysis approach. Geoderma 107:109–141. https://doi.org/10.1016/S0016-7061(01)00143-4
